# *Nannochloris* sp. Microalgae Strain for Treatment of Dairy Wastewaters

**DOI:** 10.3390/microorganisms11061469

**Published:** 2023-05-31

**Authors:** Anca Paulenco, Alin Cristian Nicolae Vintila, Alexandru Vlaicu, Mihaela Ciltea-Udrescu, Ana-Maria Galan

**Affiliations:** 1 National Institute for Research & Development in Chemistry and Petrochemistry—ICECHIM, 202 Spl. Independentei, 060021 Bucharest, Romania; anca.paulenco@icechim.ro (A.P.); mihaela.ciltea@icechim.ro (M.C.-U.); anamaria.galan@icechim.ro (A.-M.G.); 2 Faculty of Chemical Engineering and Biotechnologies, University Politehnica of Bucharest, 1–7 Polizu Street, 011061 Bucharest, Romania

**Keywords:** wastewater reclamation, cheese whey, lactose, nutrient reduction, bioactive compounds

## Abstract

This paper focuses on a process for dairy wastewater treatment by mixotrophic cultivation of microalgae *Nannochloris* sp., using cheese whey obtained as a side flow from cheese production as an organic carbon source. The microalgae samples were prepared by adding to the standard growth medium increasing amounts of cheese whey, calculated to ensure a lactose concentration between 0 and 10 g/L. The samples were incubated at a constant temperature of 28 °C and 175 rpm stirring speed for a total time of seven days. Two LED (Light Emitting Diode) illumination schemes were applied in order to assess the effect of this parameter on microalgae development and bioactive compound accumulation: continuous illumination (light stress) versus alternative cycles of 12 h light—12 h dark (day–night cycle). The growth medium was analyzed before and after microalgae cultivation in order to determine the reduction of carbon, nitrogen, and phosphorus. The results obtained for this process, after a seven-day cultivation period, were as follows: reduction of 99–100% of lactose from the growth medium, up to 96% reduction in chemical oxygen demand, up to 91% reduction in nitrogen content, and up to 70% reduction in phosphorus content.

## 1. Introduction

Research results to date have shown that biomass produced by microalgae can be significant, both quantitatively and qualitatively [1,2]. These results have generated and continue to generate more and more studies dedicated to using algal biomass as a raw material for various biotechnologies [3,4,5,6]. The range of products that can be obtained from microalgae biomass includes biofuels (oil, ethanol, methanol, biodiesel, biohydrogen, biogas, and long-chain hydrocarbon) [7,8] and different types of high added-value products, e.g., carbohydrate, protein, phycobiliproteins (food dyes)—phycocyanin and phycoerythrin, β-carotene, vitamins (e.g., A, B1, B2, B6, B12, C, E, biotin, folic acid, and pantothenic acid), sulfated polysaccharides, γ-linolenic acid (GLA), arachidonic acid (AA), eicosapentaenoic acid (EPA), docosahexaenoic acid (DHA), i.e., omega-3 fatty acid [9,10].

The most promising biotechnologies refer to the use of microalgae for the production of biodiesel, biohydrogen (using anaerobic digestion), biogas, bioethanol, bio-methanol, bio-plastics, bio-fertilizers, medicinal products, and animal feed [2,3,11]. However, even if these types of products may have certain advantages, it is essential to assess, for each biotechnology used, the economic feasibility and the impact on the environment. We note that most published results regarding the use of microalgae biomass for obtaining valuable products refer to biodiesel, which is produced by the transesterification of lipids extracted from microalgae [12,13,14,15,16,17].

Another use for microalgae systems is in wastewater treatment, where microalgae provide dissolved oxygen for bacterial degradation of organic waste, as well as causing a decrease in soluble nutrient concentrations. Microalgae biomass resulting from wastewater treatment could be transformed into co-products, for example, bio-fertilizers and raw materials for biofuels [18,19].

The dairy industry generates large volumes of wastewater with a high content of nutrients, where microalgae epuration systems can be used. Although there are several methods for treating wastewater rich in nutrients (CO_2_, N, P, etc.), conventional methods mainly require long working times, large operating space due to multiple stages of treatment, and high energy consumption [20]. Having a simple structure and a fast growth capacity, using the CO_2_ in the environment during photosynthesis [21,22], the use of microalgae is very advantageous in treating resulting residual flows. In this way, microalgae biomass rich in added-value products can be obtained while also ensuring the treatment of dairy effluents [23] used as a growth medium by microalgae strains [24,25].

Brar et al. [26] investigated the potential of microalgae species for remediation and bioenergy production when cultivated in dairy wastewater. The growth profile of different microalgae species (*Chlorella pyrenoidosa*, *Anabaena ambigua*, and *Scenedesmus abundans*) was studied at different volume ratios of dairy wastewaters to the blue-green growth medium, commonly referred to as BG-11. Following the experiments, chemical oxygen demand (COD) showed a range of variations from 62.5 to 87.5% for elimination efficiency. All microalgae strains used by Brar et al. [26] showed a good nitrate removal efficiency (85–88%), while the phosphate reduction efficiency was between 79 and 88%.

In a recent study, Kiani et al. [27] investigated the application of single culture or consortium of microalgae for the uptake of nitrogen and phosphate in the wastewater of an acid-casein factory, using *Chlorella vulgaris*, *Tetradesmus obliquus*, and *Nannochloropsis oceanica*. The consortium was found to be more effective than the respective monocultures in recovering nutrients from the wastewater, achieving 88% removal of protein while completely removing nitrate and phosphate from the effluent within 14 days. In terms of biomass production, the best result was obtained by the single culture of *Chlorella vulgaris*, followed closely by the consortium.

Mercado et al. [28] studied the feasibility of cultivating microalgae in agro-industrial wastewater as an alternative culture medium that induces the accumulation of compounds with potential bioenergetic applications. They cultivated microalgae strain *Scenedesmus* sp. for 11 days in photobioreactors (PBR), day–night cycle (12 h/12 h), both in the specific cultivation medium (BG11) as well as in 100% dairy industry wastewater (cheese and lactic derivatives production) showing a change in the biomass composition in the case of cultivation in wastewater from the dairy industry, which acts as a stress factor, increasing the concentration of lipids and decreasing the protein content. The cultivation of *Scenedesmus* sp. with 100% wastewater from the dairy industry also led to an 88% reduction in total nitrogen, 97% in total phosphorus, and 89% in COD.

The goal of this study was to demonstrate the efficiency of the *Nannochloris* sp. microalgae strain in the removal of contaminants from dairy industry wastewaters, namely cheese whey effluents, used as an organic carbon source for mixotrophic cultivation. A first element of novelty for this study is the use of the strain *Nannochloris* sp. for dairy wastewater treatment, an often-overlooked microalgae strain, despite its high lipid productivity, which could offset some of the costs [7]. Research studies that take advantage of the adaptability of microalgae towards using various carbon sources are some of the most common approaches for directing microalgae growth and changing the composition of primary metabolites [19,29]. This has been attributed to the different metabolic pathways that microalgae cells will undergo as a result of the diversity in nutrient availability in the growth medium [30]. Another novelty element is the comparison between the use of a continuous illumination system and a day–night cycle of illumination for *Nannochloris* sp. microalgae cultivation on dairy wastewaters and the effect it has on its metabolic activity.

## 2. Materials and Methods

### 2.1. Cheese Whey Preparation for Inoculum and Characterization

The cheese whey used for the experimentations was obtained from a dairy farm where cow milk is used to prepare “Telemea”, a Romanian-specific cheese similar in appearance to feta cheese. Before being used for sample preparation, the cheese whey was submitted to a pretreatment stage. It was deproteinized, by precipitation, in order to avoid inhibition of the microalgae growth process. Deproteinization was performed by maintaining the cheese whey volume at boiling temperature for 15 min, with continuous stirring, followed by gradual cooling until room temperature, to facilitate precipitation, centrifugation, and filtration of suspended solids [31]. The deproteinized cheese whey (DCW) was analyzed to determine the composition of the main contaminants considered for investigation: lactose, total nitrogen (TN), total phosphorus (TP), chemical oxygen demand (COD), as well as pH and salinity.

To determine initial lactose concentration, 0.2 mL of the sample solution was diluted up to 2 mL, followed by adding 1 mL of a 3,5-Dinitrosalicylic acid (DNSA) reagent. For determining the final lactose concentration, 2 mL of the sample solution was taken, followed by the addition of 1 mL of the DNSA reagent. The mixture was heated in a hot water bath at 95 °C for 5 min and then immediately cooled using cold water. The solution was analyzed using a UV-spectrophotometer (Ultra 3600 RIGOL, Romspectra)-based DNSA assay for reducing sugars performed at 540 nm [32]. Total nitrogen (TN) content was determined by the ISO/DIS 23697–2 method for water quality. Total phosphorus (TP) content was determined using standard methods for the examination of water and wastewater, edition 20, 4500-P C, vanado–molybdophosphoric acid method. Chemical oxygen demand (COD) was determined based on methods developed by the US Environmental Protection Agency (EPA 410.4/1993) approved for water and surface wastewater COD determination. The pH was measured by a Consort C931 (Consort, Belgium) instrument. Salinity was measured using a densimeter-salometer calibrated up to 30% salinity in the sample.

### 2.2. Microalgae Cultivation and Growth Conditions

*Nannochloris* sp. 424-1 is a proprietary patented microalgae strain [33], deposited in 2013 at the Culture Collection of Algae and Protozoa at the Scottish Marine Institute, Aryl, UK, under the code CCAP 251/10.

Deproteinized cheese whey (DCW), priorly characterized, was used for culture medium preparation. The standard culture medium for the microalgae strain selected for these experiments, *Nannochloris* sp., was a modified Zarrouk medium [34], which has previously been shown to yield good results both in terms of biomass production and lipid content [7,35], with a composition on nutrient salts as presented in Table 1. The final medium prepared for sample inoculation was composed of standard medium with incremental addition of DCW volumes corresponding to a final lactose concentration in the sample of 0, 2.5, 5, 7.5, and 10 g/L lactose. Samples were identified using the coding L which stands for lactose, followed by the concentration of lactose in each sample, from 0 g/L (blank, L0) to 10 g/L (L10). The corresponding volume of cheese whey added was calculated considering the lactose concentration of the DCW stock solution.

The inoculum of the *Nannochloris* sp. microalgae strain required for sample inoculation was prepared in advance in Erlenmeyer flasks, for 8 to 10 days of development time, at an ambient temperature of 21 ± 1 °C until the exponential growth phase of the microalgae strain was reached. Constant spectrophotometric monitoring of suspension samples at a wavelength of 678 nm allowed the identification of the point at which the microalgae cells pass from the inductive phase to the exponential growth phase. After reaching the exponential phase, the inoculum was used directly (without removal of the growth medium) for inoculating the control, and DCW-modified samples of various lactose concentrations, at a 1:9 (*v*/*v*) ratio of inoculum to the culture medium. The experiments were performed in triplicate, with data obtained expressed as mean value ± limits. For statistical analysis, the ANOVA (one-way analysis of variance) technique was employed to evaluate the results. Furthermore, data were analyzed to determine the statistical differences between the average measurements of independent groups by performing multiple comparison post hoc t-tests. If a threshold level of significance, *p* < 0.05, is reached, then differences are considered statistically significant.

The inoculated samples were grown for seven days in an Innova 42R benchtop incubator with a built-in shaker (New Brunswick, Eppendorf, Germany) that allows cultivation at constant temperature and also provides the possibility of sample stirring due to its built-in rotative platform. Cultivation parameters were set at 28 ± 1 °C temperature and 175 rpm, a rotation speed that maintains the microalgae cells in suspension and does not allow adhering to the flask walls without affecting their growth rate.

The illumination programs selected for microalgae growth consisted of both continuous high-light LED illumination and day–night cycle, consisting of 12 h LED illumination and 12 h of darkness, throughout the seven days of cultivation. The LED illumination was supplied by the integrated lighting system of the benchtop incubator, which provided the samples with cool white light with an average photosynthetic active radiation (PAR) of 240 μmol/m^2^∙s^1^ photons. The two illumination programs were proposed in order to determine which is more efficient for contaminant reduction, based on the consensus that the day–night illumination system allows microalgae cells to synthesize, during the high-illumination period, basic molecules, through photosynthesis, using the elements present in the nutrient medium, and in the darkness period, the cells transform the basic molecules in compounds with a higher molecular mass, such as pigments, carbohydrates, proteins, lipids [36]. 

### 2.3. Effect of Stress Factors on Biomass Productivity and Biocompound Accumulation

Microalgae harvesting was done by centrifuging each sample at 7000 rpm for 15 min, using a Rotina 420R Hettich centrifuge. The supernatant was collected separately for analysis of lactose, TN, TP, COD, pH, and salinity. After removing the supernatant, the microalgae biomass was washed with two portions of distilled water to remove residual salts remaining on the surface of the microalgae cells. The wet microalgae biomass was then freeze-dried using a Martin Christ Alpha 1–4 LDpLUS Lyophiliser, and microalgae productivity was determined for each sample.

Dried microalgae biomass samples were grinded thoroughly to achieve a better extraction yield. Weighed samples of dried biomass were submitted to three successive extractions with methanol in order to extract as much of the bioactive compound as possible. For each extraction, the dry biomass to solvent ratio used was 0.03 g to 2 mL (the equivalent of a 1/66.67 *w*/*v* ratio). Extractions were carried out overnight, at 4 °C, under stirring at 150 rpm, followed by removal of supernatant via centrifugation. For each sample, all consecutive effluents from the three successive extractions were reunited and analyzed spectrophotometrically. Absorption spectra were measured between 400 and 800 nm.

Concentrations of chlorophyll a and b, as well as carotenoids, were determined using Equations (1)–(3), published in the work of Xiong et al. [37], based on spectrophotometric analysis of the methanol extracts.

Chlorophyll a (mg/L) = 16.82 × A_665_ − 9.28 × A_652_ (1)

Chlorophyll b (mg/L) = 36.92 × A652 − 16.54 × A_665_ (2)

Carotenoids (mg/L) = (1000 × A_470_ − 1.91 × Chl_a_ − 95.15 × Chl_b_)/225 (3)

where A_x_ is the absorbance of the sample, measured at a wavelength of “x” nm, and Chl_a/b_ is the concentration of chlorophyll a/b, determined by Equations (1) and (2). 

For evaluating the lipid fraction of each microalgae biomass sample, extractions were carried out using a 2:1 (*v*/*v*) chloroform:methanol mixture, at a biomass to solvent ratio of 1:4 (*w*/*v*), based on a procedure modified from Folch et al. [38]. For quantitative and qualitative determination of fatty acids and of the lipid fractions previously extracted, a two-step transesterification process was carried out, using 4% potassium hydroxide methanolic solution at reflux for 2 h and then with 20% boron trifluoride methanolic solution at reflux for 90 min; a procedure developed by Galan et al. [39]. The ratio of biomass to the two methanolic solutions was 1:20:27 (*w*/*v*/*v*). The fatty acid methyl esters were isolated from the reaction products via liquid–liquid extraction and phase separations using hexane and a 20% sodium chloride aqueous solution as part of the organic layer, which was then oven dried and analyzed via GC-MS in order to determine their fatty acid profiles.

## 3. Results

### 3.1. Cheese Whey Characterization

Cheese whey was analyzed after deproteinization, and the average results regarding its organic load are presented in Table 2. The organic load of this waste flow is much higher than the threshold values considered suitable for waste flow release in the environment, provided by European Directive 91/271/EEC concerning urban wastewater treatment. The indicated requirements for streams released into the environment are as follows: for total nitrogen: 15 mg/L, for phosphorus: 2 mg/L, and for COD: 125 mg/L O_2_. 

The results obtained for the untreated DCW highlight the need for an efficient treatment method to be applied for this specific waste stream before it can be safely released into the environment.

### 3.2. Growth Medium Analysis after Microalgae Cultivation

The results regarding organic load reduction in the cheese whey-modified growth medium by microalgae strain *Nannochloris* sp. are presented in Table 3 for day—night illumination cycle and Table 4 for continuous illumination. These results were obtained after only seven days of microalgae strain cultivation in the nutrient-rich growth medium. Experimental sets were carried out with fresh batches of cheese whey, which was deproteinized before use. Although this choice resulted in small variations in the physical–chemical characterization of DCW, it was the preferred method to diminish the risk of contamination and spoiling. Table 3 and Table 4 also show the average volumes of DCW added to the growth medium to ensure the specified lactose concentrations. Each sample had a total volume of 200 mL, with 20 mL representing the microalgae inoculum, while the other 180 mL consisted of a standard medium mixed with DCW.

**Figure 1:**
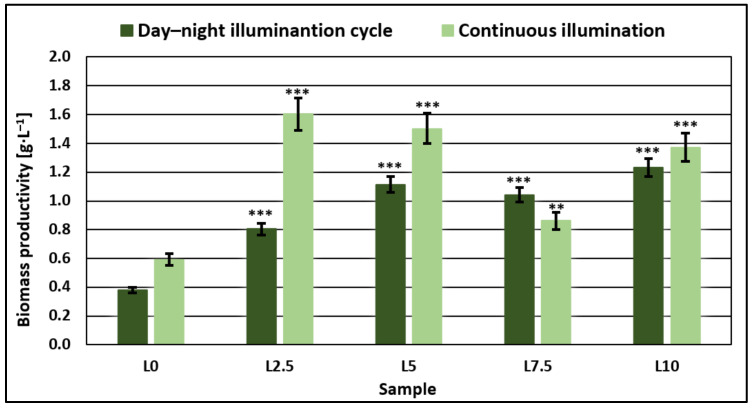
Comparison of biomass productivity values between day–night and continuous illumination systems for samples prepared with incremental concentrations of DCW in the microalgae growth medium. One-way ANOVA (*p* < 0.05) and multiple comparison post hoc *t*-tests (*n* = 5) were used to analyze the data, with asterisks indicating significant differences between control samples and each group (** = *p* < 0.01 and *** = *p* <0.001).

The best results regarding nutrient reduction were obtained for the highest concentrations of DCW in the growth medium. In all cases tested, lactose was reduced to 98–100%. Microalgae biomass production increased directly with lactose concentration in the sample (Figure 1), which in turn led to a higher reduction in other contaminants, such as nitrogen, phosphorus, and COD.

*Nannochloris* sp. is a microalgae species that has not been reported in studies regarding wastewater reclamation, yet offers great benefits in this regard, achieving a reduction in COD levels of up to 93%, up to 94% TN removal, and up to 82% TP removal, in only seven days cultivation time (continuous illumination). The addition of DCW as a carbon source allows for higher nitrogen and phosphorus reduction rates to be achieved over the control samples, regardless of the illumination system that was used. Furthermore, when factoring the combined stress, organic substrate present in the growth medium in the form of DCW as well as light stress in the form of continuous illumination, both nitrogen and phosphorus reduction rates tend to be higher than those in the day–night illumination cycle, when comparing samples with similar quantities of DCW added. To better understand these results, the microalgae biomass productivity for each sample must be considered.

### 3.3. Microalgae Biomass Productivity and Characterization

From Figure 1, we can observe that biomass productivity increased with the amount of DCW added in the growth medium, with one-way ANOVA analysis combined with multiple comparison post hoc *t*-tests (*n* = 5) indicating a significant difference for each sample over the control samples, in both types of illumination cycles. For the day–night cycle, productivity increases gradually, proportional to lactose concentration, whereas, for continuous illumination, productivity values are more than double that for the samples cultivated in the standard growth medium without added DCW. The highest biomass productivity was obtained in the continuous illumination system when the growth medium was modified through the addition of DCW corresponding to a lactose concentration of 2.5 g/L. 

With the increase in added DCW to equivalent lactose concentrations of over 5 g/L, biomass productivity was still improved over the control samples. However, this increase in productivity became less significant, especially compared to those obtained for samples with added DCW equivalent to a lactose concentration of 2.5 g/L. Additionally, with this increase in the volume of added DCW, the difference in productivity decreased between samples obtained in continuous light and those obtained in a day–night cycle, most likely as a result of the microalgae cells using the available carbon substrate for growth without relying on photosynthetic metabolic pathways.

**Figure 2:**
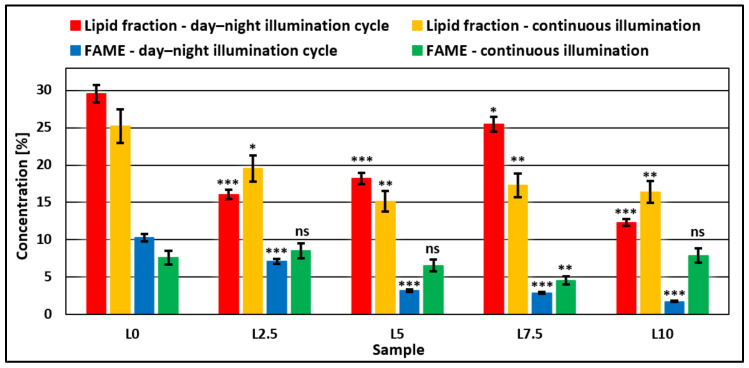
Comparison of lipid fraction and oil content expressed as methyl esters (FAME) between day–night and continuous illumination systems for samples prepared with incremental concentrations of DCW in the microalgae growth medium. One-way ANOVA (*p* < 0.05) and multiple comparison post hoc *t*-tests (*n =* 5) were used to analyze the data, with asterisks indicating significant differences between control samples and each group (* = *p* < 0.05, ** = *p* < 0.01, *** = *p* <0.001, and ns = no significant difference).

Lipid content presented an opposite trend to biomass productivity (Figure 2). The higher the biomass productivity of the sample, the lower the lipid fraction contained by the microalgae biomass. Statistical analysis, through the ANOVA technique combined with post hoc *t*-tests (*n =* 5), showed a significant decrease (*p* < 0.05) in lipid fractions for samples grown in a medium with added DCW for both types of illumination cycles. 

The same can be observed for total fatty acid concentration, with a significant decrease in FAME concentration being observed for the day–night cycle. On the other hand, for continuous illumination, statistical analysis showed no significant differences (*p* > 0.05) between microalgae samples grown with added DCW and control samples, with the exception of sample L7.5, containing added DCW equivalent to a lactose concentration of 7.5 g/L, which showed a significant decrease (*p* < 0.01). This can be explained by the fact that the microalgae strain uses the lactose and nutrients present in the medium in order to grow and multiply, considering this environment ideal for the growth process. When the microalgae strain is subjected to stress factors, such as the light stress for experiments carried out with continuous illumination, it consumes nutrients present in the growth medium and stores them in the form of lipids and pigments so as to have an emergency build-up [40].

**Figure 3:**
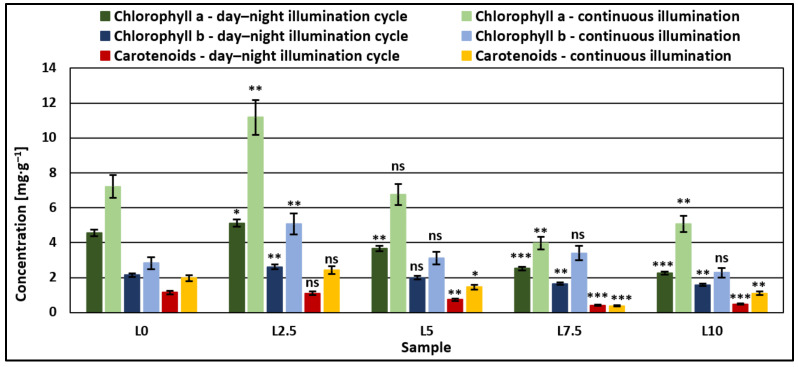
Comparison of pigment accumulation (chlorophylls and carotenoids) between day–night and continuous illumination systems. One-way ANOVA (*p* < 0.05) and multiple comparison post hoc *t*-tests (*n =* 5) were used to analyze the data, with asterisks indicating significant differences between control samples and each group (* = *p* < 0.05, ** = *p* < 0.01, *** = *p* <0.001, and ns = no significant difference).

Results for pigment accumulation (Figure 3) were generally higher in the case of continuous illumination, with values achieved for continuous illumination being more than double, in some cases, compared to the day–night cycle. Statistical analysis has shown that compared to control samples, microalgae grown in a medium with added DCW equivalent to a lactose concentration of 2.5 g/L reach significantly higher concentrations of chlorophyll a and b (*p* < 0.05), regardless of the type of illumination, while in the case of carotenoids, there are no significant changes. Increasing the amount of added DCW led to pigment concentrations decreasing visibly in most cases with the amount of DCW added to the growth medium, or in the case of chlorophyll b, showing no significant differences over the control samples. This indicated that at available lactose concentrations higher than 2.5 g/L, the microalgae strain tends to accumulate biomass (Figure 1) rather than synthesize bioactive components.

For the control samples, the unsaturated fatty acid distribution (Table 5) in the case of continuous illumination was 83%, sensibly higher than that for the day–night illumination cycle, which was 66.5%, highlighting that stress caused by constant illumination favors the production of unsaturated fatty acids. For microalgae cultivated on cheese whey, the proportion of fatty acids was generally distributed towards unsaturated fatty acids, present at around 80% of total fatty acids, with slight variations amongst the different concentrations of lactose present in the growth medium. 

The principal fatty acids identified in the lipid fractions extracted from *Nannochloris* sp. samples are palmitic acid (C16:0), oleic acid (C18:1), linoleic acid (C18:2), and linolenic acid (C18:3). With the presence of various lactose amounts in the growth medium, and considering the illumination system, the distribution shift mainly occurs amongst these four fatty acids. For the control sample (L0), the continuous illumination system aids in increasing the percentage of polyunsaturated fatty acids in the lipid fraction: C18:0 decreases, and C18:1 and C18:2 concentration increases. The shift in fatty acid composition could be a result of the stress factors present during microalgae growth, specifically light stress, which favors the metabolic pathway resulting in the transformation of C18:0 to polyunsaturated fatty acids [41].

With the increase of lactose concentration in the growth medium, the microalgae strain continues to produce higher concentrations of polyunsaturated fatty acids, mainly C18:2 and C18:3. There is also an increase in C16:2 and C16:3 concentrations compared to the day–night cycle of illumination.

## 4. Discussion

The use of microalgae strain *Nannochloris* sp. for the treatment of dairy wastewaters was proven to be efficient, both in the reduction of nutrient content in the medium and also for obtaining microalgae rich in added value compounds such as lipids (up to 25% of dry biomass), with a high proportion of unsaturated fatty acids (over 80% of total fatty acids) and considerable amounts of antioxidants, consisting in chlorophylls, up to 16 mg/g and carotenoids, up to 2.5 mg/g. The results obtained in this study are in accordance with the literature data regarding other strains of microalgae used for wastewater treatment. Plöhn et al. [42] reported that the microalgae strain *Scenedesmus quadricauda* used for dairy wastewater treatment achieved removal yields of approximately 92% nitrogen and 71% phosphates. 

Goswami et al. [43] listed in their review some removal efficiencies for microalgae used for dairy wastewater treatment, such as *Chlorella vulgaris*, with a removal rate of 85% TN, 66% TP, and 81% COD, with maximum biomass productivity of 1.23 g/L after 10 days cultivation; *Chlamydomonas polypyrenoideum*, with a removal rate of up to 90% TN; *Scenedesmus quadricauda*, with a removal rate of 86% TN, 90% TP, and 64% Total Organic Carbon, with maximum biomass productivity of 0.47 g/L after 12 days cultivation; *Tetraselmis succica*, with a removal rate of 45% TN, 42% TP, and 40% Total Organic Carbon, with maximum biomass productivity of 0.56 g/L after 12 days cultivation.

Similar research concludes that there is a relationship between nitrogen availability and lipid content in microalgae biomass. When microalgae cells are grown with nitrogen deficiency, the metabolic pathway for carbon fixation changes from protein synthesis to lipid production. It was found that the lower the initial content of nitrogen in the culture medium, the greater the number of lipids in the biomass [40]. The shift in fatty acid distribution towards unsaturated fatty acids, from 66% to over 80% in the day–night cycle, is similar to that identified in the recent literature regarding other microalgae strains grown on wastewater. Goswami et al. [43] reported data for *Chlorella vulgaris*, with 77% unsaturated fatty acids, *Scenedesmus quadricauda*, with 75% unsaturated fatty acids, and *Tetraselmis succica*, with 85% unsaturated fatty acids.

## 5. Conclusions

The present research conditions achieved parameter concentration reductions of up to 94% for nitrogen, 82% for phosphorus, and 93% for COD values—for microalgae cultivation under continuous illumination conditions, and up to 90%, 65% and 94%, respectively, for microalgae cultivation under the day–night illumination cycle. The reduction of phosphorus content was visibly lower for day–night illumination cycles. For nitrogen and COD, the threshold percentages of nutrient removal were achieved in both illumination systems and for phosphorus in continuous illumination, as mentioned by the European Directive 91/271/EEC. However, the concentrations for COD, TN, and TP after microalgae cultivation are still higher than those indicated by the Directive, even when the threshold reduction rates are achieved. For a continuous illumination growth system, for example, after cultivation in a growth medium modified by adding DCW equivalent to a lactose concentration of 10 g/L, the average values for TN, TP, and COD were 20, 60, and 900 mg/L, respectively, instead of the required 15, 2, and 125 125 mg/L, respectively (European Directive 91/271/EEC). However, given the initial high organic load, the reduction is significant. 

Further studies will focus on the potential of using multi-step microalgae-based treatment processes, with effluents not in line with requirements for release into the environment being recirculated and used for growth medium preparation of new microalgae cultivation batches. The nutrient reduction could therefore be reduced to percentages much higher than 90% [44]. For the procedure proposed in this paper, the recirculation of the growth medium can be easily integrated into the process for main waste effluent dilution before microalgae cultivation or for microalgae inoculum preparation, aiding in wastewater reclamation.

## Figures and Tables

**Table 1:** Zarrouk culture medium nutrient composition.

Component	Concentration
NaHCO_3_	16.80 g/L
K_2_HPO_4_	0.50 g/L
NaNO_3_	2.50 g/L
K_2_SO_4_	1.00 g/L
NaCl	1.00 g/L
MgSO_4_ · 7H_2_O	0.20 g/L
CaCl_2_ · 2H_2_O	0.04 g/L
Microelement solution ^1^	1 mL/L
Chelated Fe solution ^2^	5 mL/L

^1^ Micronutrient stock solution (g/L): H_3_BO_3_, 2.860; MnSO_4_ · 4H_2_O, 2.030; ZnSO_4_ · 7H_2_O 0.222; MoO_3_ (85%) 0.018; CuSO_4_ · 5H_2_O 0.079; Co(NO_3_)_2_· 6H_2_O, 0.494. ^2^ For chelated iron solution, 0.69 g FeSO_4_ · 7H_2_O and 0.93g Na_2_EDTA were dissolved in 80 mL distilled water. After a short boiling time followed by cooling at room temperature, the solution was brought to the final volume of 100 mL.

**Table 2:** Physical–chemical characterization results for DCW.

Parameter (Unit)	Value
COD (mg/L)	68,300 ± 3730
TN (mg/L)	280 ± 11
TP (mg/L)	1228 ± 46
Lactose (g/L)	61 ± 3.5
pH	6.08 ± 0.17
NaCl (%)	2.50 ± 0.1

**Table 3:** Results regarding nutrient reduction for experiments performed in day—night illumination cycle for samples prepared with incremental concentrations of DCW in the microalgae growth medium.

		Sample ID				
		L0	L2.5	L5	L7.5	L10
**Added DCW (mL)**		-	8.1 ± 0.1	16.4 ± 0.2	24.6 ± 0.3	32.8 ± 0.4
**Before** **microalgae** **cultivation**	**Lactose (g/L)**	-	2.5	5	7.5	10
	**COD (mg/L)**	-	2800 ± 14	5601 ± 37	8401 ± 58	11201 ± 81
	**TN (mg/L)**	412 ± 9	406 ± 7	401 ± 12	396 ± 11	390 ± 14
	**TP (mg/L)**	89 ± 4	136 ± 5	182 ± 4	229 ± 6	276 ± 8
**After** **microalgae** **cultivation**	**Lactose (g/L)**	-	0.0110 ± 0.0012	0.0078 ± 0.0018	0.0110 ± 0.0035	0.0249 ± 0.0059
	**COD (mg/L)**	-	385 ± 3	470 ± 5	615 ± 8	660 ± 11
	**TN (mg/L)**	290 ± 7	240 ± 6	190 ± 2	70 ± 3	40 ± 2
	**TP (mg/L)**	74 ± 3	78 ± 2	80 ± 3	94 ± 4	96 ± 5
**Parameter reduction**	**Lactose (%)**	-	99.56 ± 0.05	99.84 ± 0.036	99.85 ± 0.047	99.75 ± 0.059
	**COD (%)**	-	86 ± 0.27	92 ± 0.35	93 ± 0.33	94 ± 0.37
	**TN (%)**	30 ± 0.14	41 ± 0.12	53 ± 0.24	82 ± 0.26	90 ± 0.32
	**TP (%)**	17 ± 0.10	43 ± 0.15	56 ± 0.25	59 ± 0.23	65 ± 0.30

**Table 4:** Results regarding nutrient reduction for experiments performed with continuous illumination for samples prepared with incremental concentrations of DCW in the microalgae growth medium.

		Sample ID				
		L0	L2.5	L5	L7.5	L10
**Added DCW (mL)**		-	8.5 ± 0.1	17.1 ± 0.2	25.6 ± 0.3	34.1 ± 0.4
**Before** **microalgae** **cultivation**	**Lactose (g/L)**	-	2.5	5	7.5	10
	**COD (mg/L)**	-	3084 ± 21	6171 ± 43	9254 ± 67	12342 ± 94
	**TN (mg/L)**	412 ± 9	365 ± 4	360 ± 7	355 ± 6	350 ± 8
	**TP (mg/L)**	89 ± 4	141 ± 3	203 ± 6	264 ± 7	325 ± 7
**After** **microalgae** **cultivation**	**Lactose (g/L)**	-	0.0570 ± 0.0030	0.059 ± 0.0035	0.0640 ± 0.0115	0.1290 ± 0.0090
	**COD (mg/L)**	-	415 ± 5	400 ± 4	880 ± 9	900 ± 12
	**TN (mg/L)**	330 ± 8	180 ± 6	170 ± 3	140 ± 4	20 ± 2
	**TP (mg/L)**	78 ± 2	76 ± 3	80 ± 3	86 ± 4	60 ± 2
**Parameter reduction**	**Lactose (%)**	-	97.72 ± 0.054	98.82 ± 0.056	99.15 ± 0.053	98.71 ± 0.120
	**COD (%)**	-	87 ± 0.37	94 ± 0.41	90 ± 0.36	93 ± 0.39
	**TN (%)**	20 ± 0.22	51 ± 0.14	53 ± 0.28	61 ± 0.27	94 ± 0.36
	**TP (%)**	12 ± 0.15	46 ± 0.12	61 ± 0.29	67 ± 0.31	82 ± 0.34

**Table 5:** Fatty acid distribution in the lipid fraction extracted from microalgae biomass for samples prepared with incremental concentrations of DCW in the microalgae growth medium.

Fatty Acid	Day–Night Illumination System					Continuous Illumination System				
	L0	L2.5	L5	L7.5	L10	L0	L2.5	L5	L7.5	L10
**Palmitic acid (C16:0)**	16.35	14.71	16.00	14.62	16.04	15.19	16.51	17.90	13.03	19.85
**Palmitoleic acid (C16:1)**	4.72	3.50	7.06	4.66	5.12	5.12	2.61	5.04	4.22	6.98
**7,10 Hexadecadienoic acid (C16:2)**	7.89	14.41	8.88	7.96	12.82	12.25	15.44	12.80	11.10	5.86
**7,10,13 Hexadecatrienoic acid (C16:3)**	8.21	7.32	1.23	3.40	5.72	7.80	4.00	2.65	6.72	2.82
**Stearic acid (C18:0)**	17.22	2.00	4.85	4.02	2.38	1.77	2.89	2.21	4.11	7.21
**Oleic acid (C18:1)**	12.74	6.69	34.35	33.25	12.85	8.87	8.53	12.97	15.70	19.45
**Linoleic acid (C18:2)**	6.50	31.09	21.84	17.06	25.89	26.01	31.55	26.44	26.21	27.20
**Linolenic acid (C18:3)**	26.37	20.28	5.79	15.03	19.18	22.99	18.47	19.99	18.91	10.63
**Saturated fatty acids**	33.57	16.71	20.85	18.64	18.42	16.96	19.40	20.11	17.14	27.06
**Unsaturated fatty acids**	66.43	83.29	79.15	81.36	81.58	83.04	80.60	79.89	82.86	72.94

## Data Availability

The data that support the findings of this study are available from the corresponding author, A.M.G, upon reasonable request.
